# Differential Expression of Copper-Zinc Superoxide Dismutase Gene of *Polygonum sibiricum* Leaves, Stems and Underground Stems, Subjected to High-Salt Stress

**DOI:** 10.3390/ijms11125234

**Published:** 2010-12-17

**Authors:** Chun-Pu Qu, Zhi-Ru Xu, Guan-Jun Liu, Chun Liu, Yang Li, Zhi-Gang Wei, Gui-Feng Liu

**Affiliations:** 1 The Laboratory of Forest Genetics and Breeding and Biotechnology of Ministry of Education, Northeast Forestry University, 26 Hexing Road, Harbin 150040, China; E-Mails: qcp0451@msn.com.cn (C.-P.Q.); liuchun314@yahoo.com.cn (C.L.); liyangzaixian362@163.com (Y.L.); zhigangwe@163.com (Z.-G.W.); liuguifeng@126.com (G.-F.L.); 2 Life Science College, Northeast Forestry University, Harbin 150040, China; E-Mail: xuzhiru2003@126.com

**Keywords:** *P. sibiricum* Laxm., PS-CuZnSOD, RACE, real-time PCR, gene expression

## Abstract

In aerobic organisms, protection against oxidative damage involves the combined action of highly specialized antioxidant enzymes, such as copper-zinc superoxide dismutase. In this work, a cDNA clone which encodes a copper-zinc superoxide dismutase gene, named *PS-CuZnSOD*, has been identified from *P. sibiricum* Laxm. by the rapid amplification of cDNA ends method (RACE). Analysis of the nucleotide sequence reveals that the *PS-CuZnSOD* gene cDNA clone consists of 669 bp, containing 87 bp in the 5′ untranslated region; 459 bp in the open reading frame (ORF) encoding 152 amino acids; and 123 bp in 3′ untranslated region. The gene accession nucleotide sequence number in GenBank is GQ472846. Sequence analysis indicates that the protein, like most plant superoxide dismutases (SOD), includes two conserved ecCuZnSOD signatures that are from the amino acids 43 to 51, and from the amino acids 137 to 148, and it has a signal peptide extension in the front of the *N*-terminus (1–16 aa). Expression analysis by real-time quantitative PCR reveals that the *PS-CuZnSOD* gene is expressed in leaves, stems and underground stems. *PS-CuZnSOD* gene expression can be induced by 3% NaHCO_3_. The different mRNA levels’ expression of *PS-CuZnSOD* show the gene’s different expression modes in leaves, stems and underground stems under the salinity-alkalinity stress.

## 1. Introduction

Even when plants grow and develop under natural conditions, they are inevitably affected by environmental stresses due to their immobility [1]. This could lead to the production of a lot of reactive oxygen species (ROS), such as superoxide anion, hydrogen peroxide (H_2_O_2_), hydroxyl radical and singlet oxygen, which are harmful to intercellular components such as DNA, protein and membrane lipids. High active oxygen species (AOS) levels initiate signaling responses that include enzyme activation, gene expression, cell apoptosis and cellular damage [2]. Organisms employ an antioxidant defense system to protect themselves against the toxic effects caused by such a signal. Superoxide dismutases (superoxide: superoxide oxidoreductase, EC 1.15.1.1; SOD) are the first line of defense against the oxidative stresses by catalyzing reactive oxygen molecules to hydrogen peroxide that is consequently converted to water by catalase [3].

Superoxide dismutases (SODs) are important antioxidant enzymes that occur in virtually all oxygen-respiring organisms [4]. SODs catalyze the dismutation of superoxide (O_2_^−^) into molecular oxygen (O_2_) and H_2_O_2_ [2O_2_^−^ + 2H^+^ → O_2_ + H_2_O_2_]. Four types of SODs have been identified. Copper-zinc superoxide dismutase: The most important enzyme of the oxygen scavenging enzymes [5,6], which is closely related to anti-aging and resistance to stress in plants [6–9]. Iron SOD has been found in prokaryotes, in algae and in some higher plant chloroplasts [10]; Manganese SOD is found in prokaryotes and mitochondria; and a fourth with the coupled Ni (II/III) at the active site (Ni-SOD), which is found in the *Streptomyces genus* [11]. Copper-zinc superoxide dismutase can be divided into two forms; one is in cytosolic and the other is in chloroplastic isoenzymes. Copper-zinc superoxide dismutase in cytosolic is found mainly in cases of induced adverse environment [12,13].

Superoxide dismutase (SOD) was first isolated from bovine red blood cells by Mann and Keilin in 1938. Until now, copper-zinc superoxide dismutase has been cloned in several species of plants including rice, corn, and others [13–21]. The transgenic plants with overexpression of *SOD* gene in tobacco and alfalfa could resist cold stress, and markedly enhance antioxidant capacities [22–24].

*Polygonum sibiricum* Laxm. is a Dicotyledoneae *Polygonaceae* perennial herb. It grows in wetland, near the riverbank on saline and alkaline land. As one of the minority important halophytes grown in salinity-alkalinity areas, it is considered to be a promising species as a potential genetic resource of genetic transformation, and also can be employed as an experimental system for conducting research on salt resistance.

Salinity-alkalinity stress is one of the main abiotic stresses that restrict the development of agriculture worldwide. Compared to other abiotic salt stresses, there are limited studies on carbonate stress, though the main salt of soil, NaHCO_3_, has a severe effect on plants. The aim of the present study is to clone copper-zinc superoxide dismutase, which is located in the cytosolic in *P. sibiricum* Laxm., and to present the nucleotide sequence of copper-zinc superoxide dismutase, comparing its sequence with other known SODs from other species; and to evaluate this copper-zinc superoxide dismutase expression in leaves, stems and underground stems, when *P. sibiricum* Laxm. was induced under salinity-alkalinity stress. It is hoped to clarify the effects of *PS-CuZnSOD* in salinity-alkalinity resistance, forming a good basis for further study on the mechanisms of salinity-alkalinity stress tolerance in *P. sibiricum* Laxm.

## 2. Results and Discussion

### 2.1. cDNA Cloning, Sequencing and Bioinformatics Analysis of PS-CuZnSOD

**Figure 1.**
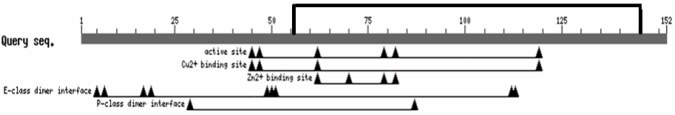

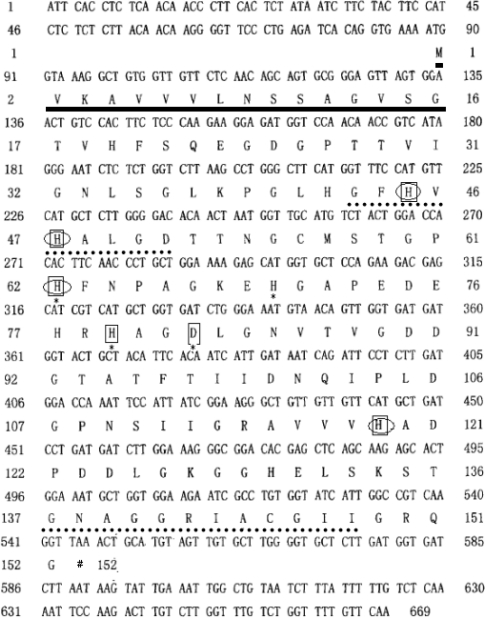
Nucleotide and deduce amino acid sequences of *PS-CuZnSOD* cDNA from *P. sibiricum* Laxm. The PCR products of *PS-CuZnSOD* cDNA were sequenced. The full length was 669 bp, with a 5′ untranslated region of 87 bp, a 3′ untranslated region of 123 bp and an open reading frame (ORF) encoding 152 amino acid (459 bp). The cDNA sequence of *PS-CuZnSOD* has been submitted to GenBank (accession No. GQ472846). The rectangle indicates the active site of the *PS-CuZnSOD*; ellipse indicates the Cu^2+^ binding site; * indicates the Zn^2+^ binding site; # indicates the stop codon. Two conserved CuZnSOD signatures are shown in broken line. The signal peptide is shown in solid line. Two cysteines (Cys56 and Cys145) form a disulfide bond for this gene.

In order to isolate cDNA encoding for copper-zinc superoxide dismutase, PCR reactions were performed using primers and total cDNA of plant leaves. Products of amplification were cloned and sequenced. Computer analysis, using the BLAST algorithm, confirmed that the selected sequence corresponded to a copper-zinc superoxide dismutase. The full-length copper-zinc superoxide dismutase cDNA fragment of *P. sibiricum* Laxm. was obtained by overlapping two cDNA fragments. The full-length copper-zinc superoxide dismutase cDNA comprised of 669 bp, containing 87 bp in the 5′-untranslated region (UTR); 459 bp in the open reading frame (ORF); and 123 bp in 3′-UTR without poly (A) tail (Figure 1). The ORF encodes a polypeptide of 152 amino acids. The calculated molecular mass of the mature protein (152 amino acids) is 15.3 kDa, with an estimated p*I* of 5.7. Two conserved CuZnSOD signatures are from the amino acids 43 to 51, and from the amino acids 137 to 148. The cDNA sequence and deduced amino acid sequence has been submitted to the NCBI GenBank as accession number GQ472846. Two cysteines (Cys56 and Cys145) form a disulfide bond for this gene.

### 2.2. Homology Comparison of PS-CuZnSOD

**Figure 2.**
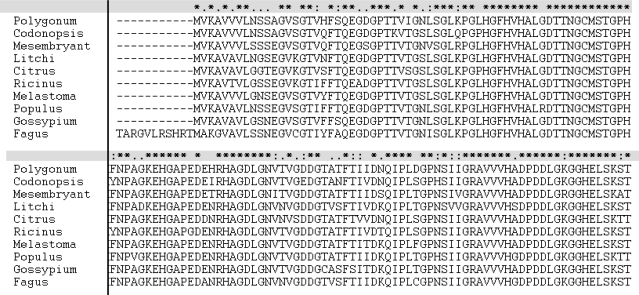
Alignment of the deduced amino acid sequences of PS-CuZnSOD and the known copper-zinc superoxide dismutase from GenBank. Identities compared with those from *Melastoma malabathricum*, *Mesembryanthemum crystallinum*, *Fagus sylvatica*, *Populus suaveolens*, *Gossypium hirsutum*, *Codonopsis lanceolata*, *Ricinus communis*, *Litchi chinensis*, *Citrus limon* were 92, 89, 83, 90, 91, 90, 89, 87 and 87 respectively.

**Figure 3.**
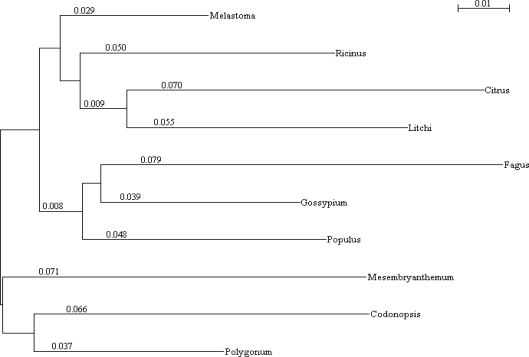
The phylogenetic tree of *PS-CuZnSOD* from plants and animals. Clustal W was used to establish the phylogenetic tree, and the result was displayed using Treeview software.

The comparison of the ORFs with other known SODs indicates that the *PS-CuZnSOD* shows homology: Identities = 125/136 (92%); with *Melastoma malabathricum*: Identities = 121/136 (89%); with *Mesembryanthemum crystallinum*: Identities = 122/147 (83%); with *Fagus sylvatica*: Identities = 122/136 (90%); with *Populus suaveolens*: Identities = 123/136 (91%); with *Gossypium hirsutum*: Identities = 122/136 (90%); with *Codonopsis lanceolata*: Identities = 120/136 (89%); with *Ricinus communis*: Identities = 117/136 (87%); with *Litchi chinensis*: Identities = 117/136 (87%); with *Citrus limon* and so it continues. *PS-CuZnSOD* was genetically distinct from other kinds of SOD (Figure 2). A phylogenetic tree based on evolutionary distances was constructed from amino acid sequences using the njplotWIN95 program (Figure 3). All of the bioinformatics analysis results suggested that *P. sibiricum* Laxm. SOD should be a plant cytoplasm copper-zinc superoxide dismutase.

### 2.3. Tissue Expression of *PS-CuZnSOD*

**Figure 4.**
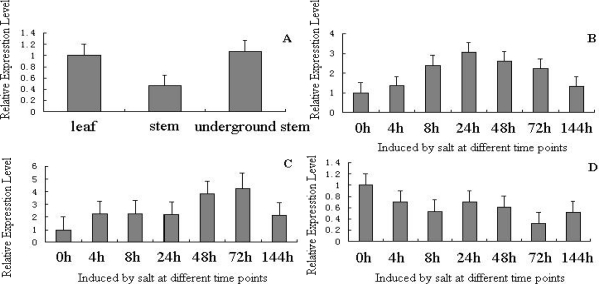
The change of *PS-CuZnSOD* after 3% NaHCO_3_ exposure in leaves, stems, underground stems’ organs. Total RNA was prepared using SDS reagent for all *P. sibiricum* Laxm. samples taken at 0, 4, 8, 24, 48, 72, 144 h, independent of 3% NaHCO_3_ stress condition. After digested with DNase I to eliminate the genome contamination, the cDNA was synthesized using the oligo d (T) primer and random 6 primer. Real-time PCR was performed with the DNA Engine Opticon™-II sequence detection system. SYBR green Real-time PCR mix (TaKaRa) was used for PCR. (**A**) The expression of *PS-CuZnSOD* gene in leaves, stems, underground stems’ organ without stress comparison; (**B**) The levels of *PS-CuZnSOD* mRNA in leaves tissues; (**C**) The level of *PS-CuZnSOD* mRNA in stems tissues; (**D**) The levels of *PS-CuZnSOD* mRNA in underground stems tissues. A multiple comparisons test was conducted to compare significant differences in *PS-CuZnSOD* expression between leaves, stems and underground stems using the SPSS software. A significant level of *p* = 0.05 was chosen.

Copper-zinc superoxide dismutase expressed in each organ of *P. sibiricum* Laxm. is shown in Figure 4. In a RT-PCR study, specific primers (SOD-F: 5′-AGTGCGGGAGTTAGTGG-3′ and SOD-R: 5′-CGATGCTCGTCTTCTGG-3′) were used to amplify a 203 bp fragment with cDNA from leaves, stems and underground stems, organs using 18S as a positive control. The RT-PCR showed that the CuZnSOD was detected in leaves, stems and underground stems. In leaves, the increase of the copper-zinc superoxide dismutase mRNA expression level reached its peak in 24 hours after 3% NaHCO_3_ stress, and gradually decreased (Figure 4B). In stems, the increase of the copper-zinc superoxide dismutase mRNA expression reached its peak in 72 hours after salinity-alkalinity stress (Figure 4C). That is, in leaves and stems they were up-regulated and then down-regulated during 3% NaHCO_3_ stress. Contrastingly, the copper-zinc superoxide dismutase transcripts were fluctuated and down-regulated after 3% NaHCO_3_ stress in underground stems’ organs (Figure 4D).

Tissue distribution of *PS-CuZnSOD* mRNA was ubiquitous in all the tissues examined in this study, which is not surprising since the expression of copper-zinc superoxide dismutase in a wide range of cell types has already been found previously. Based on experimental exposures to 3% NaHCO_3_, *P. sibiricum* Laxm. SOD mRNA was apparently affected by the durations of 3% NaHCO_3_ stress. mRNA expression of *PS-CuZnSOD* was durations-dependent in general; the saturation of expression (reaching maximum level) was observed in stems at 72 h based on the RT-PCR. There were some different responses of *PS-CuZnSOD* to 3% NaHCO_3_ exposure in every organ (or tissue) from our data. We deduce that tuning expression of copper-zinc superoxide dismutase mRNA may be used to change copper-zinc superoxide dismutase activity and in turn modulate plant growth under the salinity-alkalinity stress. The different expression mode of copper-zinc superoxide dismutase in leaves, stems and underground stems might be the reason *P. sibiricum* Laxm. has higher efficiency and economy in antioxidant and resistance to stress in plants. However, the specific factors underlying the regulatory mechanism have not been clearly understood. Our results may provide the basis for future investigations of copper-zinc superoxide dismutase roles in salinity-alkalinity stress development in different organs.

## 3. Experimental Section

### 3.1. Plant

*P. sibricum* Laxm. plants (plant height 10–15 cm) were obtained from salinity-alkalinity fields in Zhaodong, Heilongjiang (pH = 8.68). As the underground stems of *P. sibiricum* Laxm. are used for experimental materials, the experiments of rapid propagation have been carried out and the materials are grown in phytotron at 24 °C. The samples were treated at different stages using 3% NaHCO_3_. A total of 210 plants (plant height 10–15 cm) were allotted into seven treatments randomly: *i.e.*, 0 h (blank), 4 h, 8 h, 24 h, 48 h, 72 h and 144 h. Each treatment consists of six replicates with five plants of *P. sibricum* Laxm. each. After being harvested, all samples were immediately preserved in liquid nitrogen and kept at −80 °C until they were used for isolating the RNA.

### 3.2. RNA Isolation from *P. sibiricum* Laxm. and Reverse Transcription (RT)

Total RNA was extracted using a phenol sodium dodecyl sulfate extraction/LiCl precipitation procedure [25].

### 3.3. Obtaining 3′ and 5′ Regions by RACE

To isolate the complete 5′ and 3′ regions of this gene, the rapid amplification of cDNA ends (RACE) method was used. First-strand cDNA synthesis was performed using Smart™ RACE cDNA amplification kit (Clontech). Previously we obtained the *PS-CuZnSOD* 3′ EST sequences from the *P. sibiricum* Laxm. cDNA Library [26]. According to the EST, two specific primers were designed on the basis of the SOD 3′UTR for 5′-RACE. SODZnN: 3′-AGAACCAAACAGACCAAAACAAG-5′, SODZn: 3′-CTCATAACATAAGGAAAGAAAGGG-5′. The 5′ fragment PCR was then carried out according to the manufacturer’s instructions (Clontech Kit). Next, the fragments were cloned and sequenced. A pair of specific primers was designed to amplify the ORF of *PS-CuZnSOD* gene (SOD-A: 5′-GATTACAGCCAATTTCAATAC-3′, SOD-S: 5′-CTCTTACAACAAGGGGTTC-3′).

### 3.4. Subcloning

The PCR fragments were subjected to electrophoresis on 0.8% agarose gel for length differentiation, and amplified cDNA fragments were cloned into the pGEM-T Easy vector following the instructions provided (Promega, Madison, WI, U.S.). Recombinant bacteria were identified by blue/white screening and confirmed by PCR. Plasmids containing the insert were purified (Promega minipreps) and used as a template for DNA sequencing.

### 3.5. Nucleotide Sequence Analysis

The fragments were linked by the soft Bio-Edit CAP contig assembly program. The *PS-CuZnSOD* gene sequence was analyzed and compared using the BLAST P and ORF search programs with GenBank database search. The multiple sequence alignment of *PS-CuZnSO* gene was created by Clustal W analysis program, signal-peptide site was predicted by Signal P3.0, the SOD protein MW and p*I* were computed by ProtParam [27], and disulfide connectivity was predicted by SCRATCH Protein Predictor [28].

### 3.6. Quantification of *PS-CuZnSOD* Gene Expression by Real-Time RT-PCR

Total RNAs were isolated by SDS method from different tissues including stems, underground stems and leaves, at different handling stages induced by 3% NaHCO_3_, treat at 0 h (blank), 4 h, 8 h, 24 h, 48 h, 72 h and 144 h. The residue of DNA were removed by DNase I digesting, at 37 °C for 30 min. 4 microgram of the total RNA were used in each lane and electrophoresed in a 0.8% agarose gel, at 100 V/12 cm for 15 min. First-strand cDNA synthesis was performed using M-MLV reverse transcriptase (TaKaRa Biotechnology (Dalian) Co., Ltd. Japan) to transcribe poly (A)^+^ RNA with oligo-d(T)18 and random six as the primers, reaction conditions were recommended by the manufacturer’s instructions. The cDNA was used for the assay of quantitative real-time PCR. The SYBR Green I real-time RT-PCR assay was carried out in an Option-II Sequence Detection System (MJ Research, U.S.). The amplifications were performed in a 96-well plate in a 25 μL reaction volume containing 12.5 μL of 2 × SYBR Green Master Mix (TARAKA), 2.5 μL (each) SOD-F and SOD-R primers (10 mM), 1 μL of template, and 9 μL of DEPC-water. The thermal profile for SYBR Green real-time PCR was 95 °C for 2 min, followed by 45 cycles of 95 °C for 15 s and 60 °C for 30 s. In a 96-well plate, each sample was conducted in triplicate. DEPC-water for the replacement of template was used as negative control. The relative expression was calculated as 2^−ΔΔ^*^Ct^*; *Ct*: cycle threshold.

### 3.7. Statistical Analysis

A multiple comparisons (Duncan’s) test was conducted to compare significant differences in *PS-CuZnSOD* expression between leaves, stems and underground stems, using the SPSS software. A significant level of *p* = 0.05 was chosen.

## 4. Conclusions

In plants, several enzymes like SODs are involved in the release of AOSs. It is known that SOD catalyzes the rapid two-step dismutation of the toxic superoxide anion to molecular oxygen and hydrogen peroxide, through alternating reduction and oxidation of the active-site metal ion [29]. SODs play an important role in antioxidant defense pathways in response to oxidative stress [10]. In this work, the full length of a *PS-CuZnSOD* gene was isolated from *P. sibiricum* Laxm. by the rapid amplification of cDNA ends method. Analysis of the nucleotide sequence revealed that the *PS-CuZnSOD* gene cDNA clone consists of 669 bp, containing 87 bp in the 5′ untranslated region; 459 bp in the open reading frame (ORF) encoding 152 amino acids; and 123 bp in the 3′ untranslated region. The gene accession nucleotide sequence number in GenBank was GQ472846. Sequence analysis indicates that the protein, like in most others plants, CuZnSOD, includes two conserved domains from amino acid 43 to 51 and from amino acid 137 to148, which catalyzes the dismutation of superoxide (O_2_^−^) into molecular oxygen (O_2_) and H_2_O_2_. Two cysteines (Cys56 and Cys145) form a disulfide bond for this gene. *PS-CuZnSOD* has a signal peptide extension in the front of the *N*-terminus (1–16 aa), which is markedly different compared to other plants intracellular CuZnSODs.

Expression analysis by real-time quantitative PCR revealed that *PS-CuZnSOD* gene is expressed in leaves, stems and underground stems. In leaves and stems it was up-regulated and then down-regulated during 3% NaHCO_3_ stress. On the contrary, the copper-zinc superoxide dismutase transcripts fluctuated and were down-regulated after 3% NaHCO_3_ stress in underground stem organs. That is, under 3% NaHCO_3_ stress, *PS-CuZnSOD* gene expression can be induced differently. It indicates that there are different express modes in leaves, stems and underground stems. We presume that *PS-CuZnSOD* genes in leaves and stems play important roles in the process of induction by salinity-alkalinity resistance, and the down-regulation of *PS-CuZnSOD* genes may be related to the effects of H_2_O_2_-products of *PS-CuZnSOD*. Additionally, it seems that the *PS-CuZnSOD* gene does not function in salinity-alkalinity resistance in underground stem organs. Currently, we do not know the exact role *PS-CuZnSOD* genes play in *P. sibiricum* Laxm. resistance to salinity-alkalinity stress. However, differential mRNA expression of some genes in *P. sibiricum* Laxm. seems to be either “protective” or cause “division of labor” [30–34]. So far, no reports have been shown that *PS-CuZnSOD* genes are differentially expressed during the salinity-alkalinity stress processes. We propose that the *PS-CuZnSOD* “division of labor” in different organs may play an important role in *P. sibiricum* Laxm. resistance to salinity-alkalinity stress. Our results may provide the basis for future investigations of *PS-CuZnSOD* roles in *P. sibiricum* Laxm. resistance to salinity-alkalinity stress.
